# Investigation of Micromechanical Properties and Tribological Behavior of WE43 Magnesium Alloy after Deep Cryogenic Treatment Combined with Precipitation Hardening

**DOI:** 10.3390/ma14237343

**Published:** 2021-11-30

**Authors:** Adrian Barylski, Krzysztof Aniołek, Grzegorz Dercz, Piotr Kowalewski, Sławomir Kaptacz, Jan Rak, Marian Kupka

**Affiliations:** 1 Institute of Materials Engineering, Faculty of Science and Technology, University of Silesia in Katowice, 75 Pułku Piechoty Street 1A, 41-500 Chorzów, Poland; krzysztof.aniolek@us.edu.pl (K.A.); grzegorz.dercz@us.edu.pl (G.D.); slawomir.kaptacz@us.edu.pl (S.K.); jan.rak@us.edu.pl (J.R.); marian.kupka@us.edu.pl (M.K.); 2 Department of Fundamentals of Machine Design and Mechatronic Systems, Faculty of Mechanical Engineering, Wrocław University of Science and Technology, Łukasiewicza Street 7/9, 50-371 Wrocław, Poland; piotr.kowalewski@pwr.edu.pl

**Keywords:** WE43 magnesium alloy, precipitation hardening, deep cryogenic treatment, microindentation, friction, wear

## Abstract

This study investigated the micromechanical and tribological properties of WE43 alloy (Mg-Y-Nd-Zr) alloy subjected to cryogenic treatment and precipitation hardening. Microindentation tests were carried out in the range of load from 100 to 1000 mN. The introduction of deep cryogenic treatment (DCT) was shown to increase hardness and Young’s modulus, and reduce the total indentation work. As the load set during the tests increased, a gradual decrease in the measured values was observed, indicating a significant relationship between the indent size and the value of the measured parameters. Cryogenic treatment used in conjunction with precipitation hardening (after solutioning and after aging) reduces the tribological wear of the alloy. Tests have shown an almost twofold reduction in the area of the wear trace and in the volumetric wear of the alloy, as well as a more than twofold reduction in linear wear, with relatively small fluctuations in the coefficient of friction. Abrasion was the main mechanism of wear. Areas where microcutting, adhesion and plastic deformation occurred were also observed. The results indicate the significant effectiveness of the applied heat treatment in improving the service life of the WE43 alloy containing rare earth metals.

## 1. Introduction

Magnesium is one of the most common metallic elements found in the earth’s crust, estimated to be about 2% of the total mass of the earth’s crust [1]. Due to a number of beneficial properties, such as high rigidity, high strength and low density (more than four times lower than iron and two and a half times lower than titanium), it is used as a component of many alloys, mostly in the automotive and aerospace industries [1,2,3,4,5].

Magnesium alloys are also increasingly used as third-generation biomaterials, the latter showing an advantageous combination of biodegradability and bioactivity. Magnesium rare earth alloys, such as the WE43, have high biocompatibility (mechanical properties similar to the cortical bone) [6]. This is a major advantage over previous generations of biomaterials (first and second), for it allows the reduction of stress shield effects [7,8,9]. Research has also shown that magnesium alloys do not cause inflammatory reactions in the body during the biodegradation process, and magnesium itself is an essential microelement involved in human metabolism; it also occurs naturally in bone tissues [10,11,12,13]. The use of rare earth elements makes it possible to modify the properties of the alloy by means of a precipitation hardening process [14]. This leads to improved mechanical, tribological and corrosive properties. At the same time, an addition of zirconium in the WE43 improves the tensile strength and allows a grain size reduction [1,4].

Cryogenic treatment is a material modification technology known since the early half of the 19th century [15]. It is most often used as a shallow (−80 °C, CT) and deep cryogenic treatment (−196 °C, DCT) [16]. Sub-zero treatment of alloys improves both their mechanical and tribological properties, while at the same time reducing the stresses present in them. Cryogenic treatment was first applied to steels [17], allowing a 50% decrease in the manufacturing cost of machining materials [18,19]. In the case of magnesium alloys, however, DCT studies focus on alloys with aluminium and gadolinium [15,20,21,22,23,24,25,26]. There is a lack of research results in the literature concerning the effect of combining precipitation hardening with deep cryogenic treatment [16]. This applies in particular to magnesium alloys with rare earth elements. The few exceptions are two earlier papers by the authors about the WE54 alloy [27,28]. The aim of the present study was to determine the changes in micromechanical properties and tribological behavior induced by the proposed treatment. The effect of increasing the load in micromechanical tests of the WE43 alloy on the results obtained was also investigated.

## 2. Materials and Methods

Commercially available WE43 magnesium alloy (manufactured by Luxfer MEL Technologies, Manchester, UK) was used for the study. The chemical composition of the alloy as confirmed by the manufacturer’s certificate is shown in Table 1.

The alloy was supplied in the form of 1000 mm long rods, 25.4 mm (1 inch) in diameter. Disc-shaped samples with the nominal diameter of the rod and 5 mm thick were cut for the tests. Deep cryogenic treatment (DCT) was conducted in liquid nitrogen at (−196 °C) for 24 h, combining it with different stages of precipitation hardening (solution heat treatment and aging). The alloy was cooled to liquid nitrogen temperature for 1 h, and after deep cryogenic treatment, it was heated at room temperature.

Solutioning and aging were carried out in a laboratory muffle furnace, FCF-5M (Czylok, Jastrzębie-Zdrój, Poland), in the air atmosphere. The time and temperature of solutioning were set at 8 h and 545 °C during the previous tests, while the aging time was set at 24 h at 250 °C [14,27,28]. Five different variants of the state of the test material were obtained: as-delivered alloy, after deep cryogenic treatment (DCT), after solutioning and subsequent deep cryogenic treatment (S + DCT), and after precipitation hardening without (S + A) and with deep cryogenic treatment (S + DCT + A + DCT). Table 2 summarizes the different treatments of WE43 magnesium alloy.

The surfaces of the samples after heat treatment and sub-zero treatment were prepared by grinding with abrasive paper with a grit size from 320 to 4000, so as to obtain a surface roughness of Ra = 0.064 µm ± 0.006 µm. The samples were then cleaned in isopropyl alcohol using an ultrasonic washer.

Micromechanical testing was conducted using the Micro Combi Tester—MCT3 (Anton Paar, Corcelles-Cormondrèche, Switzerland) at room temperature. A Berkovich indenter with an angle of α = 65.3° ± 0.3° was used. Load-unload curves were recorded continuously for 4 different values of maximum load, *F_max_*: 100 mN, 250 mN, 500 mN and 1000 mN; the load hold and unloading time was set at 30 s, in accordance with the ISO 14577 standard [29], and the maximum load hold time was 10 s. For each sample, 24 indents were made. Hardness *H_IT_* and Young’s modulus *E_IT_* were determined using the standard Oliver-Pharr method [30,31], which uses the tangent slope to the initial part of the unload curve in the calculations. The initial part of the curve is described by the formula:

(1) $$F\left(h\right)=\alpha {\left(h-h_{f}\right)}^{m}.$$

where: *F*—indenter load, *h*—depth of indenter penetration, *h_f_*—indent depth after unloading, α—a constant comprising the modulus of elasticity and Poisson’s coefficient for the indenter material and the sample, *m*—exponent depending on the indenter geometry.

The rigidity of the system, *S*, was calculated by differentiating the equation with respect to the depth of penetration, *h*:

(2) $$S={\left(\frac{dF}{dh}\right)}_{m}$$

On this basis, the contact depth, *h_c_*, was determined using the dependence:

(3) $$h_{c}=h-\varepsilon \frac{F_{max}}{S}$$

where: *h*—depth of indenter penetration, ε—constant dependent on the indenter geometry, *F_max_*—maximum load imposed on the indenter, *S*—system rigidity.

On this basis, the relationship between the cross-sectional area of the indenter, *A_p_*, and the depth of its penetration, *h_c_*, was determined: *A_p_* = f(h).

Hardness *H_IT_*, and the reduced modulus of elasticity, *E_r_*, were calculated from formulas:

(4) $$H_{IT}=\frac{F_{max}}{A_{p}}$$

where: *F_max_*—maximum load imposed on the indenter, *A_p_*—indent area after unloading (calibrated).

(5) $$E_{r}=\frac{\sqrt{\pi }\cdot S}{2\beta \cdot \sqrt{A_{p}\left(h_{c}\right)}}$$

where: *S*—system rigidity, *β*—a correction factor associated with the indenter shape ranging from 1.0226 to 1.085, about 1.05 for a Berkovich indenter [30,31], *A_p_*—indent area after unloading, *h_c_*—contact depth.

After transforming the above relation, the Young’s modulus was calculated, *E_IT_*:

(6) $$\frac{1}{E_{r}}=\frac{1-v^{2}}{E_{IT}}+\frac{1-v_{i}^{2}}{E_{i}}$$

where: *E_IT_*, *ν*—Young’s modulus and Poisson’s coefficient for the investigated material, *E_i_*, *ν_i_*—Young’s modulus and Poisson’s coefficient for the indenter material (diamond *E_i_* = 1141 GPa, *ν_i_* = 0.07)

**Figure 1:**
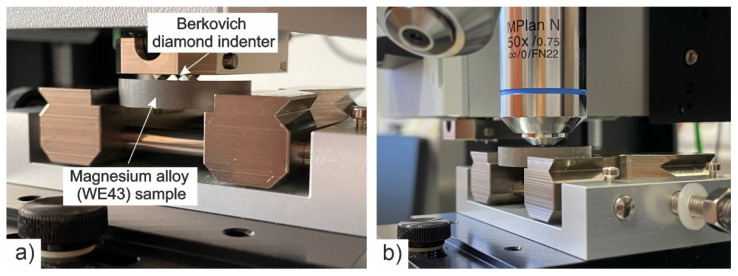
Performing an indentation measurement (**a**); microscopic observation of the indents made (**b**).

An example of a measurement of the micromechanical properties of WE43 alloy on a Micro Combi Tester (MCT^3^) is shown in Figure 1.

**Figure 2:**
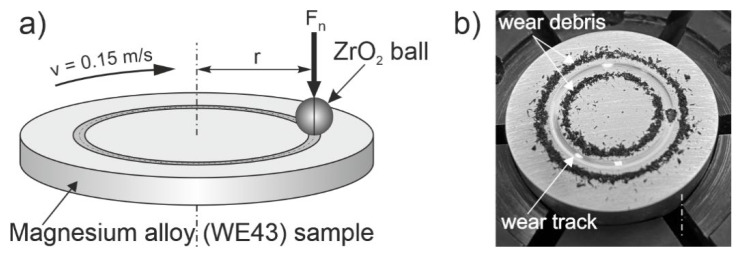
Diagram of a tribological couple (**a**); example of a WE43 magnesium alloy sample in its initial state after tribological test (**b**).

Wear tests in rotational motion were carried out on a TRN tribological tester (Anton Paar, Corcelles-Cormondrèche, Switzerland) in the ball-on-disc system (Figure 2). Test parameters were set based on previous studies [14,27,28] and the recommendations of the ASTM G99 standard [32]. The tests were performed in the following conditions:

- Load—*F_n_*: 10 (N)
- Friction distance radius—*r*: 7 (mm)
- Linear velocity—*v*: 0.15 (m/s)
- Distance—*s*: 100 (m)
- Ambient temperature: 21 ± 1 (°C)
- Air humidity: 40% ± 5 (%)

For each tested variant, 4 measurements were carried out. Ceramic balls made of zirconium dioxide, ZrO_2_, 6 mm in diameter, were used as counter-specimens.

During tribological tests the following parameters were determined: average area of the wear trace *P*; volumetric wear *V_w_*, linear wear *L_W_*, and mean friction coefficient *µ_mean_*. The coefficient of friction was recorded continuously during the tests.

Measurement of the average area of the wear trace, *P*, was made using a Form Talysurf Series 2–50 i profilometer (Taylor-Hobson, Leicester, UK). The entire area of the wear trace, i.e., 16 × 16 mm, was examined while maintaining the sampling distance of x = 10 μm, y = 50 μm. The wear trace formed during tribological tests was visualized by means of the TalyMap Universal (Version 3.2.0; Taylor Hobson Precision: Leicester, UK) and OriginPro (Version 2021. OriginLab Corporation, Northampton, MA, USA) software, acquiring an isometric 3D image of the surface studied along with a color change map. Volumetric wear, *V_W_*, was determined from the formula:

(7) $$V_{W}=\frac{V}{F_{n}\cdot s}\left(\frac{mm^{3}}{N\cdot m}\right)$$

where: *V*—volume of the wear trace of the disc determined from the formula: *V* = P·2πr (mm^3^), *P*—average area of the wear trace (mm^2^), r—radius of the friction distance (mm), *F_n_*—the load applied (N), *s*—friction distance (m).

The linear wear, *L_W_*, was measured and verified during profilometric measurements.

Observation of the microstructure and morphology of the wear traces was conducted using an Olympus GX-51 light microscope equipped with a camera and Stream Essentials software (Olympus, Tokyo, Japan), as well as a JEOL JSM-6480 scanning electron microscope (Jeol, Tokyo, Japan) equipped with an adapter for X-ray microanalysis by the EDS method (IXRF, Austin, TX, USA). Images were acquired at a magnification within the range from 30 to 4000×, which allowed observation of the microstructure after various stages of treatment and identification of the wear mechanisms occurring during friction.

## 3. Research Results and Discussion

### 3.1. Micromechanical Tests

**Figure 3:**
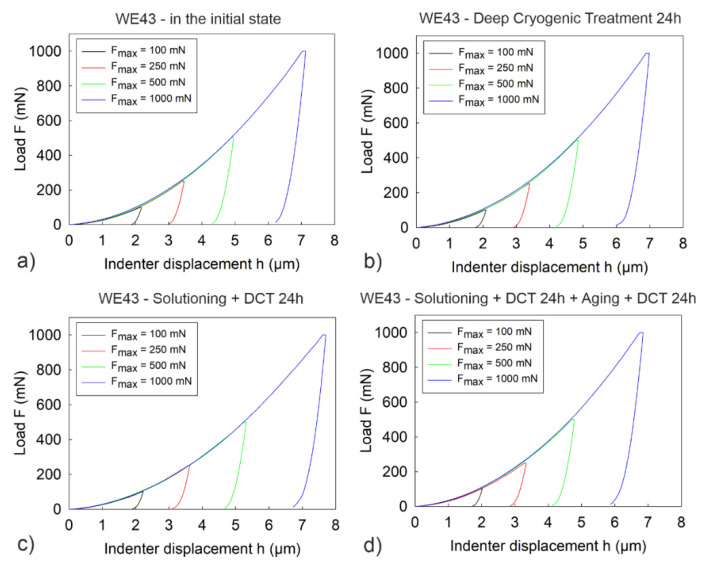
Load–unload curves *F(h)* for alloy WE43 in its initial state (**a**), after deep cryogenic treatment (**b**), after solution treatment and sub-zero treatment (**c**) and after precipitation hardening combined with deep cryogenic treatment (**d**).

Microindentation tests first determined the effect of deep cryogenic treatment (DCT) combined with precipitation hardening on the micromechanical properties of WE43 magnesium alloy and next, the effect of increasing the load applied during the tests on the measurement results was analyzed. Figure 3 shows an example of load-unload curves *F*(*h*) for different variants of the applied treatment and at different indenter loads.

**Figure 4:**
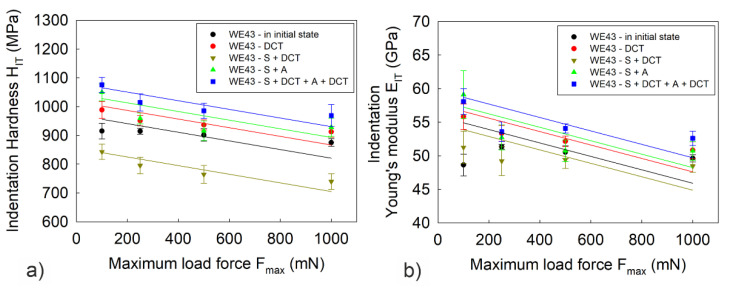
Hardness *H_IT_* (**a**) and Young’s modulus *E_IT_* (**b**) of WE43 magnesium alloy as a function of maximum indenter load *F_max_*. The error bars in the figures represent a standard deviation (σ_HIT_ = 9.97–38.26 MPa; σ_EIT_ = 0.36–3.58 GPa).

**Figure 5:**
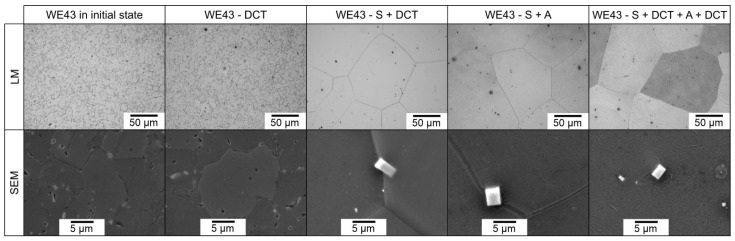
Microstructure of the WE54 magnesium alloy in the initial state, after deep cryogenic treatment (DCT), after solution treatment and deep cryogenic treatment (S + DCT), after solution treatment and aging (S + A), and after precipitation hardening combined with deep cryogenic treatment (S + DCT + A + DCT).

**Figure 6:**
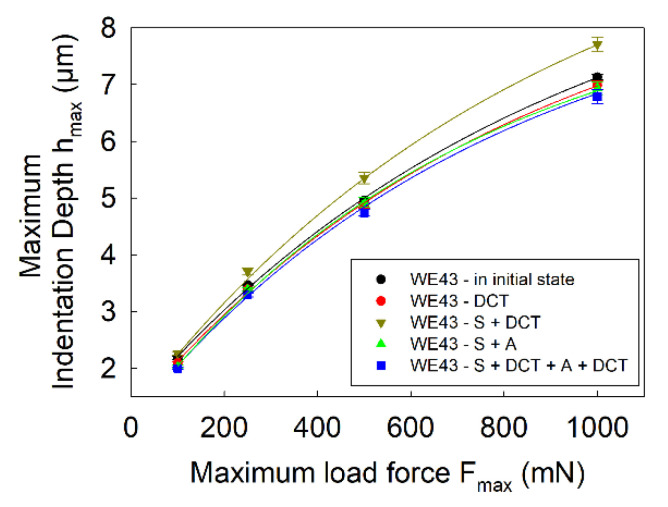
Maximum depth of indenter penetration *h_max_* during microindentation tests of WE43 magnesium alloy. The error bars represent a standard deviation (σ_hmax_ = 0.02–0.13 μm).

Figure 4, Figure 5 and Figure 6 present the dependence of hardness *H_IT_*, Young’s modulus *E_IT_*, maximum depth of indenter penetration *h_max_*, total work of indentation *W_tot_* and the percentage of the work of elastic recovery *η_IT_* on the maximum indenter load *F_max_*.

Analysis of the microindentation test results showed that the introduction of deep cryogenic treatment to the heat treatment process of the WE43 significantly improves its properties. The alloy subjected to DCT after solution heat treatment and aging was characterized by the highest hardness, *H_IT_*, and the highest Young’s modulus, *E_IT_*. An approximate 15% increase in mechanical properties was observed compared to the alloy in its initial state (Figure 4). The sub-zero treatment alone, in turn, resulted in an approximate 5–8% increase in micromechanical properties. The observed effects are the result of changes in the structure of the Mg-Y-Nd alloy studied by the authors in previous articles [27,28], where it was shown that deep cryogenic treatment caused a twofold increase in the number of *β*-phase precipitates and decrease in the grain area compared to the alloy aged without sub-zero treatment. The large number of additional precipitates formed as a result of deep cryogenic treatment after solution treatment can be explained by the formation of additional nucleation sites in magnesium alloys [15], which also occurs, among others, in magnesium alloys with aluminum and with gadolinium [20,21,22,23,24,25,26,33,34].

Changes in the microstructure after different phases of treatment observed for the WE43 alloy are shown in Figure 5. There is a clear increase in the amount of precipitates for the sample subjected to precipitation hardening combined with deep cryogenic treatment (S + DCT + A + DCT) compared to solution treatment and aging alone (S + A). The amount of precipitates produced by deep cryogenic treatment (DCT) in the precipitation hardening process is comparable to the state after doubling the aging time of a magnesium rare earth alloy without sub-zero treatment. The combination of DCT with precipitation hardening; therefore, allows the aging time to be reduced from 48 to 24 h, leading to a large reduction of processing costs.

**Figure 7:**
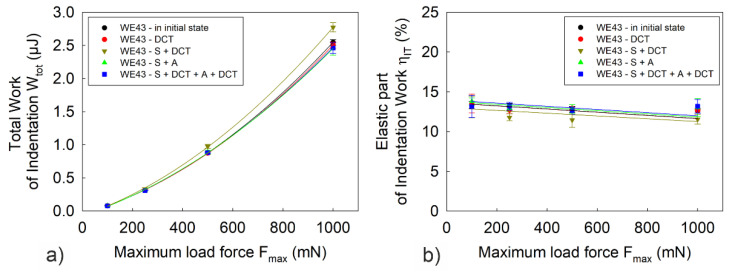
Total work of indentation *W_tot_* (**a**) and percentage of elastic deformation work *η_IT_* (**b**) of WE43 magnesium alloy as a function of maximum indenter load *F_max_*. The error bars in the figures represent a standard deviation (σ_Wtot_ = 0.002–0.094 μJ; σ_ηIT_ = 0.08–1.36%).

Observation of the load–unload curves *F(h)* for different values of maximum indenter load, *F_max_*, and the micromechanical properties determined from them (Figure 3, Figure 4, Figure 6 and Figure 7) also allowed noticing that for the WE43 magnesium alloy, there was a significant decrease in the measured quantities as the indenter load increased. This phenomenon is explained on the basis of the Taylor’s dislocation model and the relation proposed by the team of Nix, Gao [35] between the hardness, *H*, and the depth of indent, *h* (8), determined for crystalline materials and called “geometrically necessary dislocations (GNDs) model” underneath an indenter tip:

(8) $$\frac{H^{2}}{H_{0}^{2}}=\sqrt{1+\frac{h^{*}}{h}}$$

where: *H*—the hardness for a given depth of indentation *h*; *H*_0_—the hardness in the limit of infinite depth; h*—characteristic length that depends on the shape of the indenter, the shear modulus and *H*_0_.

The tests also show that, irrespective of the applied value of the maximum indenter load, the dependence of the tested quantities in relation to the applied heat treatment was maintained. In the case of hardness and Young’s modulus, the measurements showed a small scatter of results, 3% on average.

Figure 7 shows the effect of deep cryogenic treatment added to the precipitation hardening process on the variation of total indentation work, *W_tot_*, and the percentage of elastic deformation work, *η_IT_*, as a function of maximum indenter load. Analysis of the results reveals a direct correlation between the deformation resistance of the magnesium alloy studied, observed by changing parameters such as indenter penetration depth, surface area and volume of the indent. For precipitation hardening combined with DCT, the lowest values of total indentation work are observed as well as an approximately 14% share of elastic deformation work due to increase in hardness and Young’s modulus.

### 3.2. Sliding Wear Tests on WE43 Magnesium Alloy

**Figure 8:**
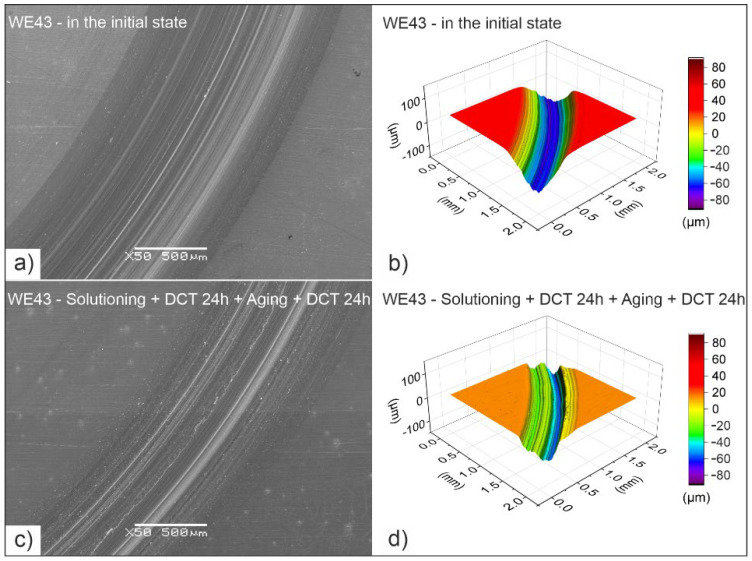
SEM microscope images and isometric 3D images of wear traces of WE43 magnesium alloy in the initial state (**a**,**b**) and after deep cryogenic treatment combined with precipitation hardening (**c**,**d**).

**Figure 9:**
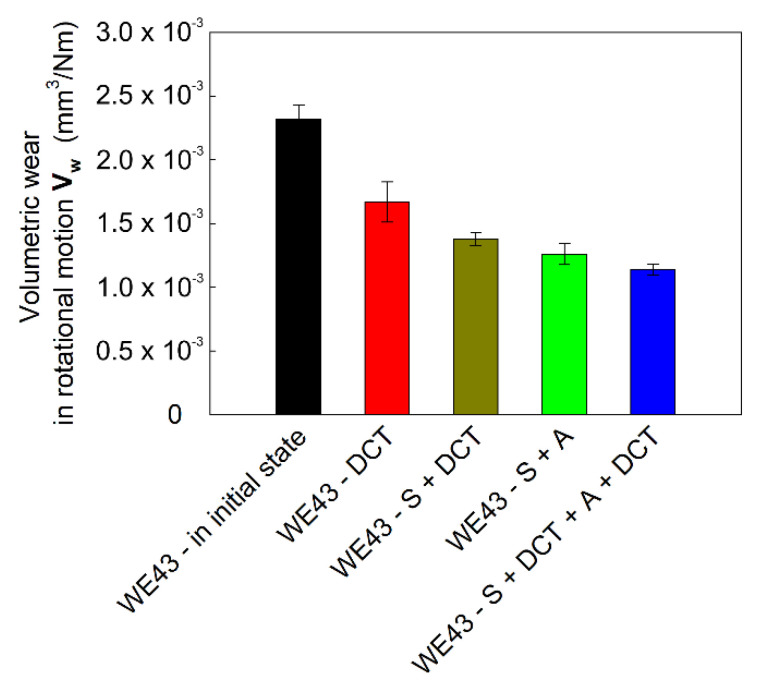
Volumetric wear *V_w_* of WE43 alloy in the initial state and after different heat treatment variants. The error bars represent a standard deviation (σ_Vw_ = 0.43 × 10^4^–1.57 × 10^4^ mm^3^/Nm).

**Figure 10:**
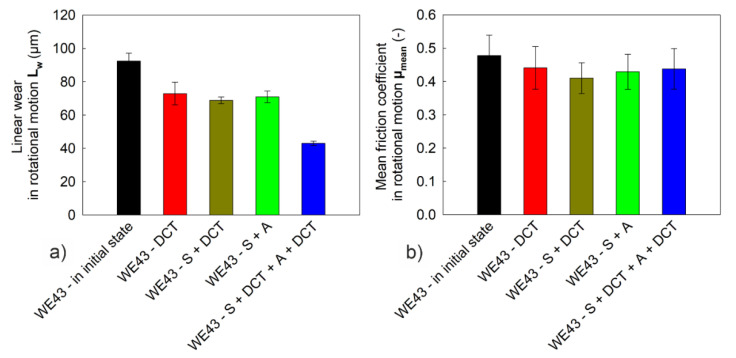
Linear wear, *L_w_*—(**a**) mean stabilized friction coefficient, *μ_mean_*—(**b**) of WE43 alloy in the initial state and after different heat treatment variants. The error bars in the figures represent a standard deviation (σ_Lw_ = 1.32–6.82 μm; σ_μmean_ = 0.046–0.064).

The consequence of the improvement in the micromechanical properties of WE43 magnesium alloy induced by sub-zero treatment and precipitation hardening are changes in tribological properties. Figure 8 presents SEM microscope images and examples of isometric 3D images from profilometric measurements of wear traces obtained in rotary motion, and Figure 9 shows the volumetric wear of the alloy, V_w_, calculated on their basis after different heat treatment variants. Figure 10 presents the linear wear, L_w_, and the mean stabilized friction coefficient, μ_mean_, of the investigated alloy.

The tribological tests that were carried out by the authors showed that the magnesium alloy in its initial state was characterized by the worst tribological properties (the largest surface area of the wear trace and the volumetric wear calculated on its basis, *V_w_* = 2.32 × 10^−3^ mm^3^/Nm) (Figure 8 and Figure 9). The application of deep cryogenic treatment alone allows for a 30% reduction in wear of the alloy, *V_w_*, while the combination of deep cryogenic treatment with precipitation hardening reduces the volumetric wear of the WE43 alloy by more than double to 1.14 × 10^−3^ mm^3^/Nm.

The analysis of the results linear wear *L_w_* (Figure 10a) confirms a significant reduction of this type of wear for the WE43 alloy subjected to sub-zero treatment at −196 °C/24 h, after solution treatment at 545 °C/8 h, and after aging for 24 h. The alloy in the initial state was characterized by linear wear of *L_w_* = 92.4 μm, while the treatment that was carried out allowed for reducing this value by more than 53%, to 43 μm. The mean stabilized coefficient of dry friction, *μ_mean_*, in rotary motion for the friction couple ZrO_2_ ball/WE43 alloy (Figure 10b) oscillated around *µ_mean_* = 0.48 for the samples in the initial state. Lower values were recorded for samples after sub-zero treatment and after solution treatment combined with sub-zero treatment, *µ_mean_* = 0.41, and *µ_mean_* = 0.44 after sub-zero treatment combined with precipitation hardening. The above results corroborate that increasing the amount of *β*-phase lamellar precipitates by introducing deep cryogenic treatment to the precipitation hardening process effectively reduces the tribological wear of the investigated alloy. Similar observations can be found in the literature in works on magnesium alloys with aluminium [16,21] and gadolinium [22,23,24,25,26] additions, and in our previous papers concerning WE54 magnesium alloy [27,28].

**Figure 11:**
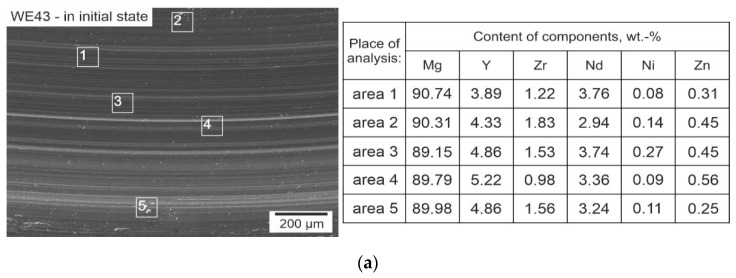

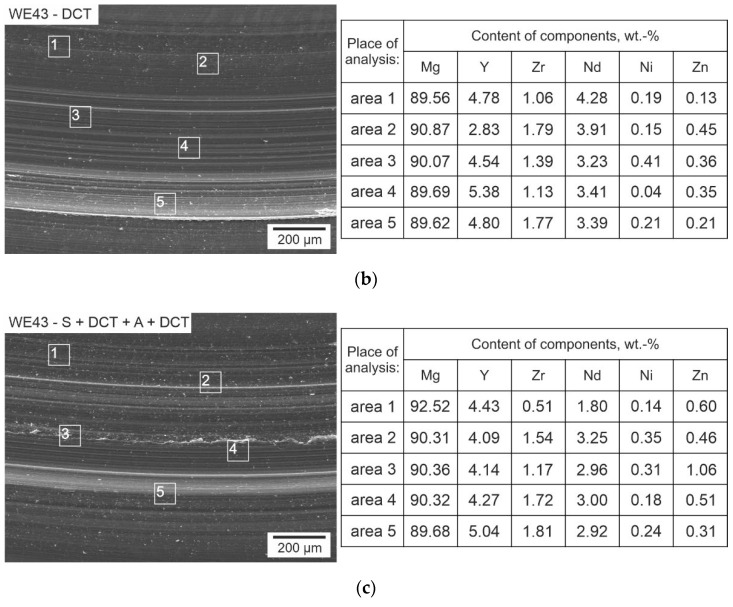
Microanalysis of the chemical composition of wear traces of the as-delivered WE43 alloy—(**a**); after deep cryogenic treatment—(**b**) and after sub-zero treatment combined with precipitation hardening—(**c**).

The results of microanalysis of the chemical composition (EDS) of the wear traces of the WE43 alloy shown in Figure 11 confirm that the applied heat treatment and the friction process itself did not have a significant influence on the change of the alloy composition. Moreover, no deposition of wear products of the counterpartners, i.e., the ZrO_2_ balls, was observed on the surface, which was confirmed by the absence of their wear observed during profilometric and microscopic measurements. The analysis results are consistent with the alloy certificate provided by the manufacturer, Luxfer Mel Technologies.

### 3.3. Determination of Wear Micromechanisms of WE43 Alloy in Rotary Motion

**Figure 12:**
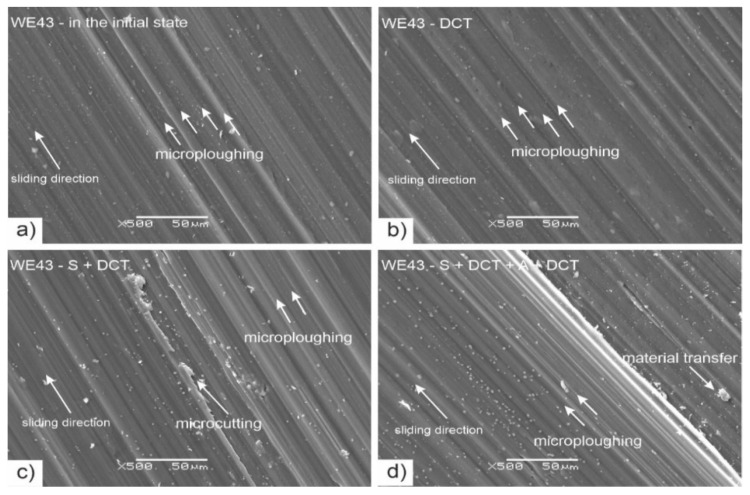
SEM images of the wear traces formed in rotary motion of as-delivered WE43 alloy—(**a**); after deep cryogenic treatment—(**b**); after solution treatment combined with DCT—(**c**) and after sub-zero treatment combined with precipitation hardening—(**d**).

**Figure 13:**
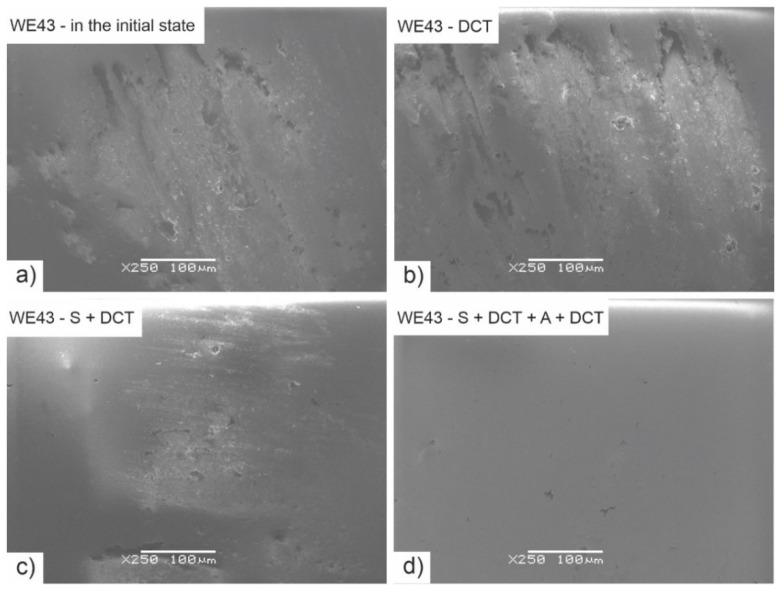
SEM images of ZrO_2_ balls after tribological tests in a pair with as-delivered WE43 alloy—(**a**); after deep cryogenic treatment—(**b**); after solution treatment combined with DCT—(**c**) and after sub-zero treatment combined with precipitation hardening—(**d**).

Figure 12 shows the results of examination of the wear trace morphologies (after dry friction in rotary motion) of WE43 magnesium alloy performed on a scanning electron microscope (SEM). Figure 13 presents SEM images of the surface of ZrO_2_ balls interacting with the studied alloy during tribological tests.

Observation of the wear traces (Figure 12) of the WE43 alloy allows for concluding that during wear in rotary motion, abrasive wear is the dominant wear mechanism. The images show numerous grooves and depressions parallel to the direction of sliding (microploughing). After solution treatment, areas of microcutting of the alloy can also be observed. Deep cryogenic treatment introduced to the precipitation hardening procedure effectively reduces this process. The images also show adhesion formed as a result of the detachment of wear particles from the alloy surface, which were displaced and reattached to the alloy. Profilometric investigations and observation of SEM images allow also to notice that DCT combined with precipitation hardening significantly reduces the process of deep scratches formation. The wear tracks are much smaller in comparison with WE43 alloy in the as-delivered condition. These mechanisms do not differ from the alloy with a higher yttrium content studied in our previous articles [14,27,28]. Similar findings can be found in the literature on magnesium–aluminum alloys, such as AE42, AZ91 [33,36] or Mg–1.5 Zn–0.15 Gd [22].

Microscopic observation after tribological testing was also performed on the ZrO_2_ counter-specimens, on which no wear was observed. However, areas of material transferred from the tested WE43 magnesium alloy samples are visible (Figure 13a–c). Deep cryogenic treatment combined with precipitation hardening also facilitates reduction of this process (Figure 13d). The absence of wear of the ZrO_2_ balls is mainly due to the large difference in hardness of the materials tested.

## 4. Conclusions

The paper presents the results of micromechanical and tribological studies, and the analysis of wear mechanisms occurring during the process of dry friction in rotary motion of magnesium alloy with a 4% yttrium content (WE43), after deep cryogenic treatment and after various stages of precipitation hardening combined with sub-zero treatment. The research carried out by the authors allows the following conclusions to be reached:

1. Deep cryogenic treatment (DCT) combined with precipitation hardening by changing the structure effectively improves the micromechanical and tribological properties of alloy WE43. Among others, a more than 15% increase in hardness *H_IT_* and Young’s modulus *E_IT_*, as well as a change in parameters such as maximum indenter penetration depth, surface area and indent volume were demonstrated. The lowest values of total indentation work *W_tot_* were observed with an about 14% share of elastic deformation work *η_IT_*.
2. As the maximum indenter load *F_max_* increased, a considerable decrease in the micromechanical properties (*H_IT_*, *E_IT_*) was observed, which indicates a strong effect of the increase in the surface area of the indents made on the WE43 magnesium alloy on the values measured by means of microindentation. The measurements showed a small scatter of results, 3% on average, and dependence of the tested quantities on the applied heat treatment was preserved for all loads.
3. The tribological tests and the parameters tested, such as: volumetric wear *V_w_*, linear wear *L_w_* and stabilized friction coefficient *μ_mean_*, indicate a twofold improvement in wear resistance of WE43 magnesium alloy subjected to deep cryogenic treatment in combination with precipitation hardening, compared to the alloy in the as-delivered condition.
4. Profilometric studies, microscopic observation and microanalysis of the chemical composition (EDS) showed that the proposed treatment (DCT + precipitation hardening) is effective in reducing the area (depth and width) of the wear traces of the magnesium-rare earth alloy and reduces the cutting process as well as the adhesion of alloy material to counter-specimens, i.e., ZrO_2_ balls, The heat treatment applied and the friction process itself have no significant effect on the change in alloy composition.
5. The examination of the morphology of the wear traces allows for the conclusion that abrasive wear was the main wear mechanism of the WE43 alloy. The SEM images showed phenomena characteristic of this wear mechanism, such as microploughing, microcutting and adhesion.
6. Further research is being conducted to understand the exact mechanism affecting the improvement of properties of magnesium alloys with rare earth metals under deep cryogenic treatment. The combination of deep cryogenic treatment and precipitation hardening is an effective method to improve the service life of WE43 magnesium alloy.

## Figures and Tables

**Table 1:** Chemical composition of the as-delivered alloy WE43.

Content of Components, wt.-%							
Y	Nd	Zr	Zn	Mn	Cu	RE	Mg
4.0	2.3	0.49	0.01	0.02	0.002	3.0	residue

**Table 2:** Summary of heat treatment applied to WE43 alloy.

Sample	Heat Treatment Applied			
	Solution Treatment	Deep Cryogenic Treatment	Aging	Deep Cryogenic Treatment
WE43 in initial state	-	-	-	-
WE43–DCT	-	−196 °C/24 h	-	-
WE43–S + DCT	545 °C/8 h	−196 °C/24 h	-	-
WE43–S + A	545 °C/8 h	-	250 °C/24 h	-
WE43–S + DCT + A + DCT	545 °C/8 h	−196 °C/24 h	250 °C/24 h	−196 °C/24 h

S—solution treatment; A—aging treatment; DCT—Δ deep cryogenic treatment.

## Data Availability

The data presented in this study are available on request from the corresponding author.
